# Blood Cells Count Derived Inflammation Indexes as Predictors of Early Treatment Response to Dupilumab in Patients with Moderate-to-Severe Atopic Dermatitis

**DOI:** 10.3390/jcm12062104

**Published:** 2023-03-07

**Authors:** Angelo Zinellu, Federica Sucato, Viviana Piras, Gian Mario Addis, Gabriele Biondi, Maria Antonia Montesu, Arduino A. Mangoni, Ciriaco Carru, Pietro Pirina, Panagiotis Paliogiannis, Alessandro G. Fois, Rosanna Satta

**Affiliations:** 1 Department of Biomedical Sciences, University of Sassari, 07100 Sassari, Italy; 2 Department of Medicine, Surgery and Pharmacy, University of Sassari, 07100 Sassari, Italy; 3 Dermatology Unit, Department of Medical Sciences and Public Health, AOU Cagliari, 09123 Cagliari, Italy; 4 Dermatology Unit, Department of Medical Care of San Francesco Hospital, 08100 Nuoro, Italy; 5 Discipline of Clinical Pharmacology, College of Medicine and Public Health, Flinders University, Adelaide 5042, Australia; 6 Department of Clinical Pharmacology, Flinders Medical Centre, Southern Adelaide Local Health Network, Bedford Park, Adelaide 5042, Australia

**Keywords:** atopic dermatitis, blood cell count, IgE, platelet-to-lymphocyte ratio, dupilumab, treatment response

## Abstract

Derived inflammatory indexes from routine hematological parameters might be useful for predicting early-response vs. late/non-response to dupilumab, the first biological agent approved for moderate-to-severe atopic dermatitis (AD). We tested this hypothesis by retrospectively investigating the association between pre-specified baseline inflammatory indexes and dupilumab response (≥50% reduction in the Eczema Area and Severity Index, EASI 50) at 4 and 16 weeks in a consecutive series of 66 AD patients (38 males and 28 females). Forty-six patients (69.7%) were early-responders at 4 weeks, whereas the remaining twenty (30.3%) were late/non-responders at 16 weeks. In logistic regression, the platelet-to-lymphocyte ratio (PLR) was independently associated with early-response (OR = 1.0159, 95% CI 1.0005 to 1.0315, *p* = 0.0426). The predictive performance of PLR and other derived indexes towards early-response was further improved by their combination with serum IgE concentrations, with a maximum AUC value for the combined systemic immune inflammation index (SII)-IgE of 0.797 (95% CI = 0.677 to 0.884, *p* < 0.0001). Derived inflammatory indexes, particularly SII-IgE, might be useful to identify early-responders to dupilumab and develop alternative treatment protocols for late/non-responders.

## 1. Introduction

Atopic dermatitis (AD), a chronic inflammatory skin disease affecting approximately 7–10% of adults and 20% of children in western countries [1], is characterized by dry skin, pruritus and eczema with recurrent flares and a negative impact on physical function and work productivity [2,3,4]. Mild forms of AD are managed with emollients and topical anti-inflammatory agents, while more severe disease requires systemic treatment with immunosuppressive drugs such as methotrexate, cyclosporin, and azathioprine. However, the systemic use of immunomodulators is not always effective and might cause significant toxicity [5]. Recently, a new class of drugs, biological agents, has been introduced to target the key inflammatory mediators involved in the pathophysiology of AD [6,7]. Dupilumab, a fully human monoclonal antibody targeting interleukin (IL)-4 and IL-13 by inhibiting their signaling through the blockade of the IL-4 receptor α subunit, is the first biological agent approved by the Food and Drug Administration (FDA) and the European Medicine Agency for moderate-to-severe AD [8,9]. The efficacy and safety of dupilumab have been demonstrated both in clinical trials [10,11,12] and in real-life cohorts [13,14,15,16]. However, the observed inter-individual variability in efficacy would benefit from the identification of specific patient characteristics predicting early-response vs. late/non-response. This information would be useful in order to provide the patient with a more precise timeframe required for disease control and, potentially, stimulate further research to identify alternative treatment protocols and regimens for late/non-responders. To date, however, such predictors have been poorly investigated.

Routinely-derived blood cell inflammation indexes are gaining interest as they reflect the systemic inflammatory burden and may predict treatment response in specific disease states. In particular, several studies have highlighted the role of specific inflammation indexes derived from neutrophils, platelets, lymphocytes, and monocytes, e.g., the neutrophil-to-lymphocyte ratio (NLR) and the platelet-to-lymphocyte ratio (PLR) as biomarkers of disease, prognosis, and treatment response [17,18,19,20,21,22]. Furthermore, the derived neutrophil-to-lymphocyte ratio (dNLR) has been shown to have a similar prognostic ability to that of the NLR in some diseases, e.g., breast cancer, COVID-19, and acute coronary syndrome [23,24,25], and can also predict the efficacy of immunotherapy in non-small cell lung cancer patients [26]. The lymphocyte-to-monocyte ratio (LMR) has been shown to independently predict clinical outcomes in soft tissue sarcoma patients receiving pazopanib treatment [27]. The aggregate inflammation systemic index (AISI) and the systemic inflammation index (SII) have been shown to have significant associations with progression-free survival in patients in advanced non-small-cell lung cancer treated with nivolumab [28], while the systemic inflammation response index (SIRI) is a useful prognostic marker in patients with advanced gastric cancer receiving neoadjuvant chemotherapy. Therefore, we sought to investigate the association between such biomarkers and early-response vs. late/non-response to dupilumab, defined as a ≥50% reduction in the Eczema Area and Severity Index score (EASI 50), in AD patients.

## 2. Methods

We retrospectively studied a consecutive series of 66 AD patients commenced on dupilumab at the Dermatology Units of the University Hospital of Sassari, the Department of Medical Sciences and Public Health, AOU Cagliari, and the Department of Medical Care of San Francesco Hospital, Nuoro, between January 2020 and December 2021.

Disease severity was assessed using EASI [29], the peak pruritus numerical rating scale (PP-NRS), and the peak sleep numerical rating scale (PS-NRS). Quality of life was evaluated using the Danish version of the Dermatology Life Quality Index (DLQI) questionnaire [30]. Patients were considered eligible for dupilumab treatment if they were unresponsive to cyclosporin or had contraindications to its use. All patients had received topical steroid and immunomodulatory treatments and systemic steroids since disease onset. All treatment was discontinued at the time of enrollment, except for skin emollients and moisturizers, with a wash-out period of four weeks.

Patients were assessed at baseline and after 4- and 16-week treatment with 300 mg dupilumab every second week following a loading dose of 600 mg, as per product information. Treatment response was defined as a ≥50% reduction in the EASI score (EASI 50) [31]. Early-responders were those exhibiting a response to dupilumab at 4 weeks. The remaining patients were defined as late/non-responders at 16 weeks.

The following demographic and clinical parameters were collected at baseline: age, sex, family history of AD, age of AD onset, and comorbidities including allergic symptoms affecting the eye and nose (allergic rhinoconjunctivitis) and the airways (asthma and rhinitis). We also assessed the following simple blood cell count inflammation parameters: white blood cell count (WBC), monocytes, lymphocytes, neutrophils, platelets, red cell distribution width (RDW), and mean platelet volume (MPV). We then extrapolated the following derived indexes: NLR (neutrophil/lymphocyte ratio), dNLR [neutrophils/(white blood cells—neutrophils)], PLR (platelet/lymphocyte ratio), LMR (lymphocyte/monocyte ratio), SIRI [(neutrophils × monocytes)/lymphocytes), SII [(neutrophils × platelets)/lymphocytes] and AISI [(neutrophils × monocytes × platelets)/lymphocytes]. Finally, we collected information regarding baseline plasma IgE concentrations.

Data are expressed as mean values (mean ± SD) or median values (median and IQR). Variable distribution was evaluated using the Kolmogorov–Smirnov test. The Student’s *t*-test or the Mann–Whitney rank sum test were used to evaluate between-group differences of continuous variables. The Fisher test or the chi-squared test were used to assess differences between categorical variables. Relationships between variables were assessed by using the Pearson’s correlation or the Spearman’s correlation as appropriate. A *p*-value < 0.05 was considered statistically significant. The parameters that were significantly associated with early-response (PLR, SIRI, AISI, SII, and IgE) were then combined by multiplying each of their values with IgE concentrations (PLR-IgE, SIRI-IgE, AISI-IgE, SII-IgE) in order to investigate their potentially superior predictive ability when compared to PLR, SIRI, AISI, SII, and IgE taken singly. SIRI-IgE and AISI-IgE values were further multiplied by a factor of 10^−3^ to facilitate data analysis and presentation. The predictive performance of the studied parameters and the optimal cut-off points for sensitivity and specificity were estimated by receiver operating characteristics (ROC) curve analysis and the Youden Index. A multivariate logistic regression analysis, using the “enter method” command, was performed to evaluate independent associations between the studied parameters and early-response vs. late/non-response status. In order to avoid collinearity bias, the independent predictive ability of each index was individually evaluated by adjusting for confounders with a *p*-value < 0.2 in univariate analysis (age at diagnosis, sex, and late-onset). Statistical analyses were performed using MedCalc for Windows, version 20.014—64 bit (MedCalc Software, Ostend, Belgium).

## 3. Results

A total of 66 AD patients [38 males and 28 females; median age at diagnosis, 6 (IQR: 1–21) years] were included in the study (Table 1). Forty-six patients (69.7%) were early-responders at 4 weeks whereas the remaining twenty (30.3%) were late/non-responders at 16 weeks. Barring IgE concentrations, there were no significant differences between early-responders and late/non-responders in clinical and demographic characteristics and simple haematological parameters (Table 1 and Table 2).

By contrast, the PLR (median: 133; IQR: 1,101–172 vs. 101; IQR: 85–124, *p* = 0.048), SII (median: 492; IQR: 367–795 vs. 366; IQR: 268–443, *p* = 0.030), SIRI (median: 0.99; IQR: 0.76–1.26 vs. 0.71; IQR: 0.52–1.06, *p* = 0.035) and EASI (median: 269; IQR: 163–375 vs. 158; IQR: 123–256, *p* = 0.025) were significantly higher in early-responders than late/non-responders. No significant between-group differences were observed in NLR, dNLR and LMR values (Table 3).

No correlation was observed between baseline EASI and simple or derived inflammation indexes, except for LMR (rho = −0.29, *p* = 0.02). A non-significant correlation trend was also observed between baseline EASI and monocyte count (rho = 0.21, p = 0.08).

ROC curve analysis was performed to evaluate the sensitivity, specificity, and accuracy of derived inflammation indexes in identifying early-responders vs. late/non-responders (Table 4). AUC values were significant for PLR (0.653), SII (0.670), SIRI (0.664), AISI (0.675), and IgE (0.776). The combined indexes, particularly the SII-IgE, further improved performance (AUC 0.797, 95% CI = 0.677 to 0.884, *p* < 0.0001; 87% sensitivity and 60% specificity using 31 as cut-off value). In multivariate logistic regression (Table 5), IgE (OR = 1.0005, 95% CI 1.0001 to 1.0009, *p* = 0.0204), combined PLR-IgE (OR = 1.0044, 95% CI = 1.0006 to 1.0083, *p* = 0.0237), SII-IgE (OR = 1.0012, 96% CI = 1.0002 to 1.0022, *p* = 0.0215), SIRI-IgE (OR = 1.9994, 95% CI = 1.1364 to 3.5170, *p* = 0.0168) and AISI-IgE (OR = 1.0023, 95% CI = 1.0003 to 1.0044, *p* = 0.0290) were significantly associated with early-response after correction for age at diagnosis, sex, and late onset.

## 4. Discussion

In March 2017, the FDA approved dupilumab as the first injectable biological for the treatment of refractory moderate-to-severe AD in view of its superior efficacy and favorable safety profile compared to conventional therapy [32]. Recently, baricitinib (a Jak1/2/3-selective inhibitor) and upadacitimib (a Jak1/2-selective inhibitor) have also been approved for the treatment of AD in some countries and will become more widely available. Jak inhibitors target the pathways involved in the pathogenesis of AD more specifically than conventional immunomodulators such as cyclosporin and azathioprine, with higher efficacy in controlling skin eruption and pruritus [33]. In this context, the availability of biomarkers of early-response to dupilumab may assist with (a) providing AD patients with a more precise timeframe required for disease control, and (b) the development of adapted dose and frequency protocols to achieve response in late/non-responders.

Systemic inflammation is a common feature of AD. Routine blood tests and derived inflammatory indexes to characterize inflammatory status are helpful in the early diagnosis, risk stratification, and prediction of treatment response of various diseases [16,17,18,19,20,21,22,23,24,25,26,27,28]. Associations between such indexes and dermatological conditions have been recently reported. In particular, the NLR and PLR have been found to be associated with the presence of psoriasis and AD [22,34,35].

The present study tested the hypothesis that blood-cell-derived inflammation indexes at baseline predict early-response at 4 weeks vs. late/non-response at 16 weeks to dupilumab in AD patients. Baseline PLR and the combination of PLR, SIRI, SII, and AISI with IgE were significantly associated with early treatment response at 4 weeks. The predictive performance of IgE-combined indexes was particularly high, with the highest AUC value observed with SII-IgE (0.797).

To our knowledge, this is the first study that reports significant associations between baseline routinely blood-cell-derived inflammation indexes and early dupilumab response in real-world AD patients. Few studies have investigated predictors of dupilumab response in this group. Olesen et al. [36] reported that younger age, female sex, a lower baseline EASI score, lactate dehydrogenase (LDH), and IgE were significantly associated with early-response. The observation regarding serum IgE concentrations is in line with our findings. Kato et al. reported that baseline serum LDH was negatively correlated with the percentage reduction in EASI score at 3, 6, and 12 months after initiating dupilumab, but not at 1 month [37]. In agreement with our data, Ferrucci et al. reported that patients with early-onset AD showed good response to treatment [38]. In this retrospective study, the absence of eosinophilia was also associated with drug response. Baseline absence of allergic conjunctivitis, history of allergic asthma, younger age, baseline PP-NRS score, and early-onset AD were identified as independent predictors of drug response by Nettis et al. [39]. In contrast, no predictive markers of response were identified by Fargnoli et al. at 4 and 16 weeks [31].

Important limitations of our study include the small size of the cohort and the retrospective design. On the other hand, the involvement of different centers with real-world experience of new treatments ensures the generalizability of our results. Pending confirmatory studies, the use of combined inflammatory indexes that also incorporate IgE concentrations might be particularly useful in the identification of late/non-responders to dupilumab who might benefit from alternative biologics or adapted dupilumab protocols for the management of AD. In this context, newly derived haematological indexes that include other cell types, e.g., eosinophils, critically involved in the pathophysiology of AD, and/or specific eosinophil-related biomarkers offer additional opportunities for further research to enhance the capacity to predict treatment response in this patient group.

## Figures and Tables

**Table 1:** Demographic and clinical characteristics of early-responders and late/non-responders.

	Total (*n* = 66)	Early-Responders (*n* = 46)	Late/No-Responders (*n* = 20)	*p* Value
Age at diagnosis (years)	6.5 (1–23)	4 (1–16)	22 (2–52)	0.06
Age when commencing dupilumab (years)	30 (22–49)	30 (22–47)	32 (23–54)	0.35
Late-onset (>18 years) (no/yes)	44/22	35/11	11/9	0.09
Male/Female	38/28	24/22	14/6	0.18
EASI at baseline	36 (26–45)	34 (26–49)	38 (26–40)	0.76
EASI at 4 weeks	14 (7–20)	10 (5–15)	25 (16–30)	<0.0001
EASI at 16 weeks	5 (2–12)	5 (2–10)	10 (4–17)	0.11
Family history of AD (no/yes)	26/40	18/28	8/12	0.95
PS-NRS score at baseline	10 (8–10)	9.5 (8–10)	10 (8–10)	0.85
PP-NRS score at baseline	9 (8–10)	9 (8–10)	8.5 (7.5–10)	0.57
DLQI at baseline	21.5 (12–29)	24 (13–29)	20 (11–29)	0.56
Asthma (no/yes)	32/34	21/25	12/8	0.29
Rhinitis (no/yes)	31/35	21/25	10/10	0.75
IgE (KU/L)	1090·(302–4260)	2315·(625–5000)	286·(84–1145)	0.0004
Previous systemic immunosuppressive treatment (no/yes)	44/22	30/16	14/6	0.71
Previous systemic steroid treatments (no/yes)	11/55	6/40	5/15	0.23
Previous systemic antihistamine treatment (no/yes)	4/62	2/44	2/18	0.38

DLQI: Danish version of the Dermatology Life Quality Index; EASI: Eczema Area and Severity Index; PP-NRS: peak pruritus numerical rating scale: PS-NRS: peak sleep numerical rating scale; AD: atopic dermatitis.

**Table 2:** Baseline simple haematological parameters in early-responders and late/non-responders.

	Total (*n* = 66)	Early-Responders (*n* = 46)	Late/No-Responders (*n* = 20)	*p* Value
RBC (×10^12^ L)	4.98 (4.61–5.40)	4.93 (4.62–5.26)	5.01 (4.41–5.48)	0.88
RDW (%)	13.2 (12.5–14.0)	13.1 (12.6–14.0)	13.5 (12.3–14.3)	0.83
PLT (×10^9^ L)	253 (221–300)	257 (229–300)	228 (207–283)	0.12
MPV (fL)	8.8 (8.0–9.5)	8.8 (8.2–9.5)	8.4 (7.8–9.2)	0.41
Neutrophils (×10^9^ L)	3.60 (2.87–4.70)	3.75 (2.93–5.12)	3.30 (2.60–4.60)	0.34
Lymphocytes (×10^9^ L)	2.13 (1.60–2.80)	2.03 (1.60–2.78)	2.34 (1.75–2.80)	0.35
Monocytes (×10^9^ L)	0.50 (0.40–0.70)	0.50 (0.40–0.72)	0.44 (0.40–0.63)	0.42
Eosinophils (×10^9^ L)	0.40 (0.20–0.62)	0.40 (0.21–0.60)	0.30 (0.20–0.70)	0.55

MPV: Mean platelet volume; PLT: platelets; RBC: red blood cells; RDW: Red distribution width.

**Table 3:** Baseline combined haematological parameters in early-responders and late/non-responders.

	Total (*n* = 66)	Early-Responders (*n* = 46)	Late/Noresponders (*n* = 20)	*p* Value
NLR	1.66 (1.33–2.46)	1.82 (1.40–2.70)	1.44 (1.24–1.87)	0.09
dNLR	1.10 (0.90–1.50)	1.11 (0.94–1.62)	1.07 (0.88–1.25)	0.24
LMR	3.97 (3.41–5.33)	3.76 (3.25–4.84)	4.39 (3.53–6.14)	0.10
PLR	122 (91–169)	133 (101–172)	101 (85–124)	0.048
SII	437 (308–629)	492 (367–795)	366 (268–443)	0.030
SIRI	0.94 (0.62–1.20)	0.99 (0.76–1.26)	0.71 (0.52–1.06)	0.035
AISI	247 (140–341)	269 (163–375)	158 (123–256)	0.025

AISI: aggregate inflammation systemic index; dNLR: derived neutrophil-to-lymphocyte ratio; LMR: lymphocyte-to-monocyte ratio; NLR: neutrophil-to-lymphocyte ratio; PLR: platelet-to-lymphocyte ratio; SII: systemic inflammation index; SIRI: systemic inflammation response index.

**Table 4:** Receiver operating characteristics (ROC) curves and prognostic accuracy of individual and combined haematological parameters.

	AUC	95% CI	*p*-Value	Cut-Off	Sensitivity (%)	Specificity (%)
IgE	0.776	0.656 to 0.869	<0.0001	>2864	48	95
PLR	0.653	0.526 to 0.766	0.040	>126	57	80
SII	0.670	0.543 to 0.780	0.025	>454	59	80
SIRI	0.664	0.537 to 0.776	0.028	>0.896	63	70
AISI	0.675	0.549 to 0.785	0.022	>164	74	60
PLR-IgE	0.792	0.675 to 0.882	<0.0001	>31	87	60
SII-IgE	0.797	0.677 to 0.884	<0.0001	>140	85	65
SIRI-IgE (×10^−3^)	0.782	0.663 to 0.874	<0.0001	>2.493	43	100
AISI-IgE (×10^−3^)	0.790	0.672 to 0.881	<0.0001	>754	44	100

IgE: immunoglobulin E; PLR: platelet-to-lymphocyte ratio; SII: systemic inflammation index; SIRI: systemic inflammation response index.

**Table 5:** Odds ratio, for laboratory parameters, obtained by multivariate logistic regression analysis.

	OR	95% CI	*p*-Value
Age at diagnosis	1.0079	0.9613 to 1.0569	0.7439
Gender	2.3905	0.6642 to 8.6031	0.1823
Late-onset	0.2941	0.0287 to 3.0130	0.3026
IgE	1.0005	1.0001 to 1.0009	0.0204

Age at diagnosis	0.9817	0.9395 to 1.0258	0.4099
Gender	1.2923	0.3661 to 4.5622	0.6903
Late-onset	0.4661	0.0584 to 3.7203	0.4714
PLR	1.0121	0.9987 to 1.0258	0.0764

Age at diagnosis	0.9800	0.9368 to 1.0253	0.3815
Gender	1.2689	0.3456 to 4.6587	0.7197
Late-onset	0.4362	0.0536 to 3.5491	0.4379
SII	1.0018	0.9995 to 1.0040	0.1266

Age at diagnosis	0.9765	0.9316 to 1.0235	0.3213
Gender	1.9113	0.5815 to 6.2824	0.2860
Late-onset	0.6167	0.0764 to 4.9817	0.2642
SIRI	1.6215	0.6941 to 3.7883	0.2345

Age at diagnosis	0.9806	0.9377 to 1.0256	0.3925
Gender	1.7984	0.5391 to 5.9991	0.3397
Late-onset	0.5486	0.0693 to 4.3420	0.5695
AISI	1.0010	0.9989 to 1.0031	0.3453

Age at diagnosis	1.0075	0.9602 to 1.0572	0.7599
Gender	2.0025	0.5524 to 7.2591	0.2906
Late-onset	0.2787	0.0261 to 2.9766	0.2904
PLR-IgE	1.0044	1.0006 to 1.0083	0.0237

Age at diagnosis	1.0073	0.9596 to 1.0573	0.7698
Gender	1.6487	0.4511 to 6.0251	0.4496
Late-onset	0.2454	0.0227 to 2.6461	0.2469
SII-IgE	1.0012	1.0002 to 1.0022	0.0215

Age at diagnosis	1.0059	0.9588 to 1.0553	0.8101
Gender	2.1116	0.5830 to 7.6488	0.2550
Late-onset	0.2855	0.0276 to 2.9487	0.2927
SIRI-IgE	1.9994	1.1364 to 3.5170	0.0168

Age at diagnosis	1.0007	0.9557 to 1.0477	0.9777
Gender	1.7420	0.4906 to 6.1853	0.3906
Late-onset	0.3586	0.0389 to 3.3019	0.3652
AISI-IgE	1.0023	1.0003 to 1.0043	0.0290

AISI: aggregate index of systemic inflammation; IgE: immunoglobulin E; PLR: platelet-to-lymphocyte ratio; SII: systemic immune-inflammation index; SIRI: systemic inflammation response index.

## Data Availability

Anonymized data are available upon reasonable request to correspond author.
